# Perforated Appendicitis Presenting as a Soft Tissue Infection of the Thigh: An Example of Successful Non-Operative Management

**DOI:** 10.7759/cureus.35560

**Published:** 2023-02-27

**Authors:** Shray Malik, Joy E Harris, Ranveer Vasdev, Anthony T Rezcallah

**Affiliations:** 1 Surgery Integrated Care Community, Minneapolis Veterans Affairs Health Care System, Minneapolis, USA; 2 College of Medicine, University of Minnesota Twin Cities, Minneapolis, USA

**Keywords:** soft tissue thigh infection, non-operative management, myositis, retrocecal appendix, perforated appendicitis

## Abstract

Perforated appendicitis is a rare but serious clinical scenario typically requiring urgent surgical intervention. Herein, we discuss the case of a 62-year-old woman with COVID-19 and ruptured retrocecal appendicitis presenting as a right lower extremity soft tissue infection that was successfully managed using non-operative measures. This unique case illustrates the feasibility of conservative care - rather than urgent surgical intervention - in the treatment of an atypical presentation of complicated appendicitis in a high-risk patient.

## Introduction

Acute appendicitis is the most common reason for emergent surgical intervention [1]. Patients classically present with a history of vague periumbilical discomfort, which later localizes to the right lower quadrant. Associated symptoms include anorexia, nausea, and vomiting. Fever and leukocytosis often develop subsequently. However, this presentation can vary significantly depending on the anatomic position of the appendix [2], resulting in delayed treatment if symptoms go unrecognized. Consequently, the appendix has been referred to as the “Great Masquerader” [3]. One anatomic variant that can present with atypical symptoms is a retrocecal appendix. Thigh pain has been documented by a number of case reports as the presenting symptom of perforated retrocecal appendicitis in both adults [4] and children [5,6]. Specific etiologies of thigh pain included cellulitis [4,7], thigh abscess [8], subcutaneous emphysema [9,10], and necrotizing fasciitis [11]. The proximity of a retrocecal appendix to the posterior abdominal wall can result in the development of a retroperitoneal abscess with inflammation extending into the thigh [12]. The majority of the reported cases were treated operatively - especially if the patient became acutely ill or septic. This case report describes an unusual presentation of acute appendicitis that was successfully treated without surgical intervention.

## Case presentation

A 62-year-old woman with a past medical history of fibromyalgia presented to the emergency department with four days of worsening right-sided thigh pain. She had a mechanical fall on her right side two weeks prior. Following that episode, she developed nausea, intermittent fevers, diaphoresis, loss of appetite, and worsening of her chronic diarrhea with several episodes of stool incontinence. She did not report vomiting, abdominal pain, melena, or hematochezia. Her acute-onset right thigh pain progressively worsened and was refractory to over-the-counter pain medications. She was eventually unable to move her right lower extremity due to pain and had significant difficulty with activities of daily living. This loss of function prompted her to call emergency medical services. She was transported by ambulance to our facility.

**Figure 1:**
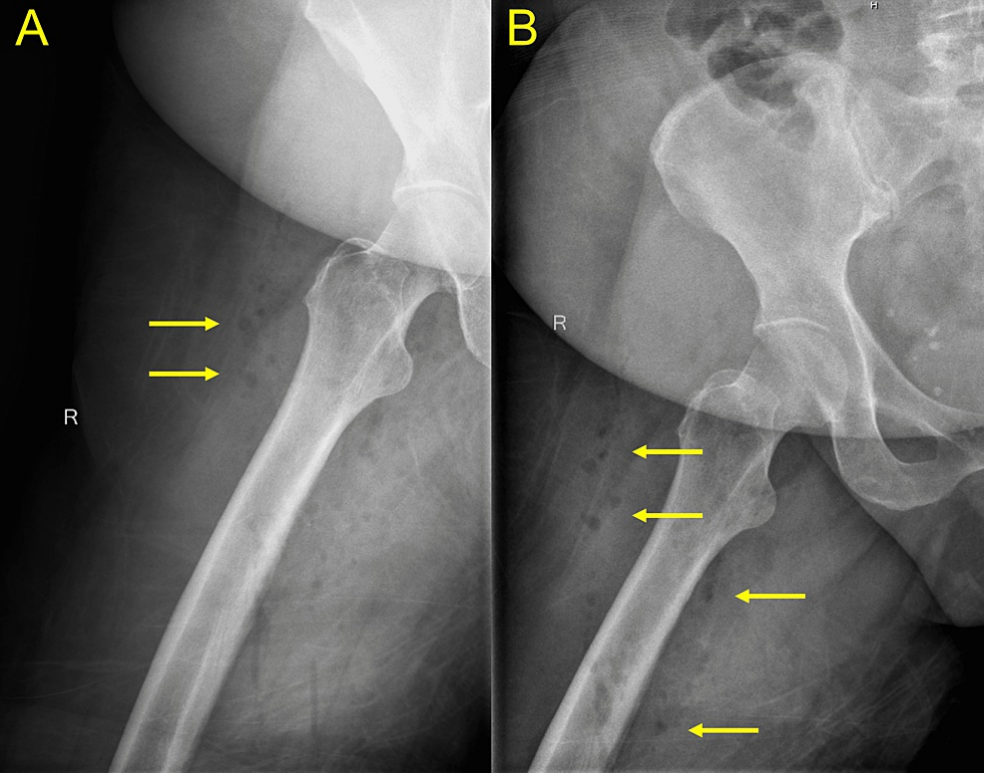
Standard roentgenography of the right lower extremity Plain films of the right lower extremity demonstrate gas in the deep tissues of the right thigh (yellow arrows). (A) Lateral view. (B) Anteroposterior view.

Her vital signs in the emergency department were notable for a temperature of 98.6° F (37.0° C), heart rate of 130 beats per minute, blood pressure of 133/85 mmHg, respiratory rate of 18 breaths per minute, and oxygen saturation of 92% on room air. Her BMI was 39.3 kg/m^2^. On examination, she had tenderness as well as extensive erythema of the right groin and anterior thigh, measuring approximately 15 by 20 cm. There was no streaking erythema, induration, fluctuance, crepitus, bullae, or drainage. Her abdomen was soft, non-tender, and non-distended without rebound or guarding. Laboratory testing revealed an elevated white blood cell (WBC) count of 13.92×10^9^ cells/L (ref: 4.5 - 11.0×10^9^ cells/L), C-reactive protein of 369.32 mg/L (ref: 3 - 10 mg/L), and lactate of 5.84 mmol/L (ref: 0.5 - 2.2 mmol/L). The patient was incidentally found to be positive for COVID-19 but did not report a cough, sore throat, or shortness of breath. Plain films of the right lower extremity revealed gas deep in the right thigh (Figures 1A, 1B), and general surgery was consulted with concern for necrotizing fasciitis. She was promptly started on intravenous piperacillin/tazobactam (3.375 g Q6H), clindamycin (900 mg Q8H), and intravenous fluids in the emergency department. Despite the imaging findings, the patient’s clinical picture did not appear consistent with a necrotizing soft tissue infection, and conservative management was continued.

**Figure 2:**
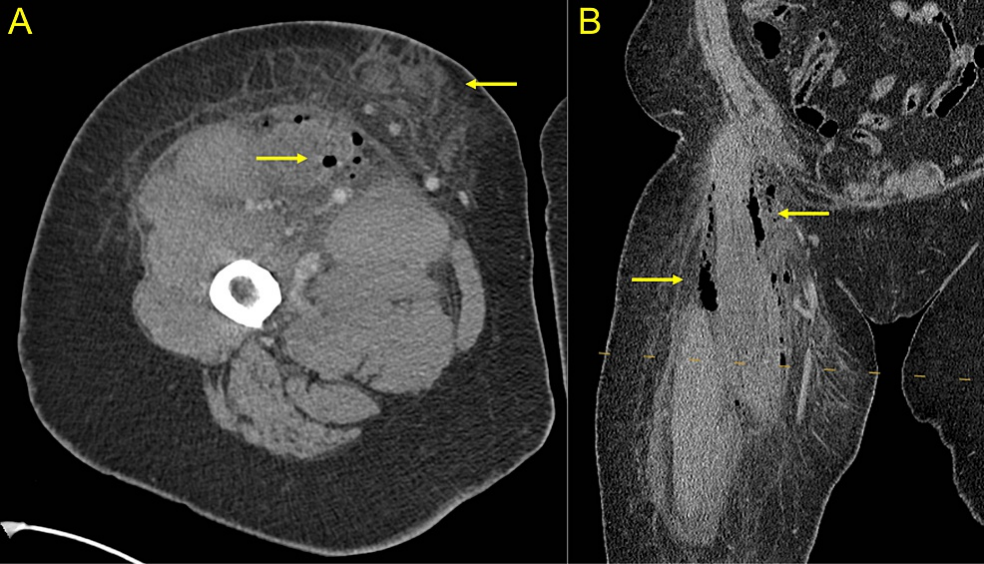
Computed tomography (CT) of the right lower extremity CT scan of the right lower extremity reveals soft tissue gas and edema (yellow arrows). (A) Axial view. (B) Coronal view.

**Figure 3:**
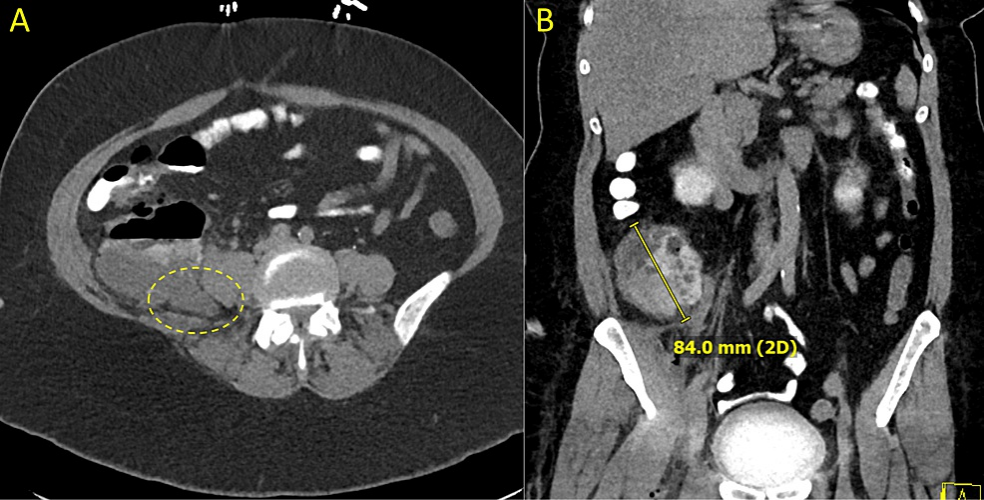
Computed tomography (CT) of the abdomen (A) Axial view demonstrating extensive inflammatory change consistent with ruptured retrocecal appendicitis (dotted circle). (B) Coronal view reveals a large intraluminal cecal abscess.

A CT scan of her right lower extremity revealed extensive fascial stranding and soft tissue gas centered around edematous musculature, concerning soft tissue infection and myositis with a gas-forming organism (Figures 2A, 2B). The right lower quadrant was partially visualized in this study and revealed extensive inflammatory changes obscuring the tip of the appendix. A CT abdomen demonstrated loculated intraperitoneal gas around the cecum extending posteriorly into the retroperitoneum and along the right iliacus muscle, as well as an intraluminal cecal abscess measuring 8.4 cm (Figures 3A, 3B). These findings were suggestive of perforated retrocecal appendicitis with inflammatory fistulization into the right retroperitoneum and right lower extremity. Orthopedic surgery was consulted due to the extensive abscesses traversing multiple joints and involving multiple muscle compartments. Subsequent MRI did not show evidence of right hip septic arthritis. Given the inflammatory process adjacent to the femoral vessels, vascular surgery was also consulted to aid in decision-making regarding possible surgical management.

Due to her morbid obesity and COVID-19-positive status, it was felt that extensive operative intervention for perforated retrocecal appendicitis would carry a significant risk of morbidity and mortality. Because the patient was clinically and hemodynamically stable, the multidisciplinary surgical team continued to recommend non-operative management with a frequent reassessment of signs and symptoms, which aligned with the patient’s preference to avoid an operation. The infectious disease team was consulted, and per their recommendations, vancomycin (1000 mg Q12H) was added to broaden antibiotic coverage.

The following morning, her lower extremity erythema had receded approximately 5 cm, and her WBC count and lactate both decreased. No bullae or streaking erythema were noted. However, she was mildly hypotensive and tachycardic, so we elected to observe her in the ICU with serial examinations. Her vital signs normalized with conservative management. Interventional radiology placed a percutaneous drain into the right lower quadrant retroperitoneal space for source control. Initially, there were no drainable fluid collections in the right lower extremity. In subsequent weeks, she developed intermittent fevers and elevated WBC counts while remaining hemodynamically stable. Fluid collections in the right lower extremity were identified on CT, and four additional percutaneous drains were placed. She underwent multiple needle aspirations of several abscesses in the right lateral thigh and medial calf. One superficial abscess in the right proximal anteromedial thigh spontaneously opened, and the patient agreed to a bedside incision to facilitate drainage and allow for wound packing. Serial imaging studies were performed to monitor for additional or expanding fluid collections.

Blood cultures grew *Parvimonas micra,* and abscess cultures grew *Escherichia coli*, *Streptococcus anginosus*, *Candida albicans*, and mixed anaerobic microorganisms. Antimicrobial treatment was narrowed to fluconazole (400 mg QD) and intravenous ampicillin/sulbactam (3 g Q6H), which was later switched to oral amoxicillin/clavulanic acid (875 mg Q12H).

**Figure 4:**
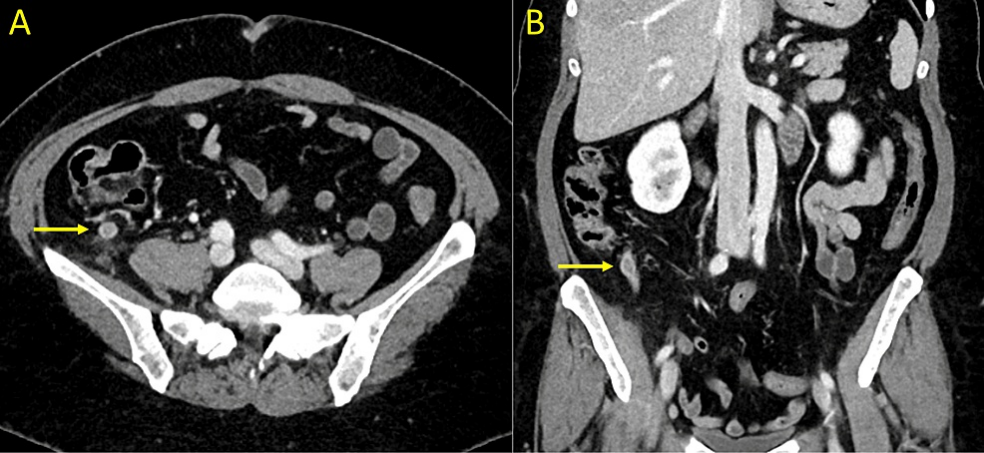
Three-week follow-up computed tomography (CT) of the abdomen CT scan of the abdomen at the patient's three-week follow-up visit revealed a 2 cm walled-off collection abutting the tip of the appendix (yellow arrows). (A) Axial view. (B) Coronal view.

**Figure 5:**
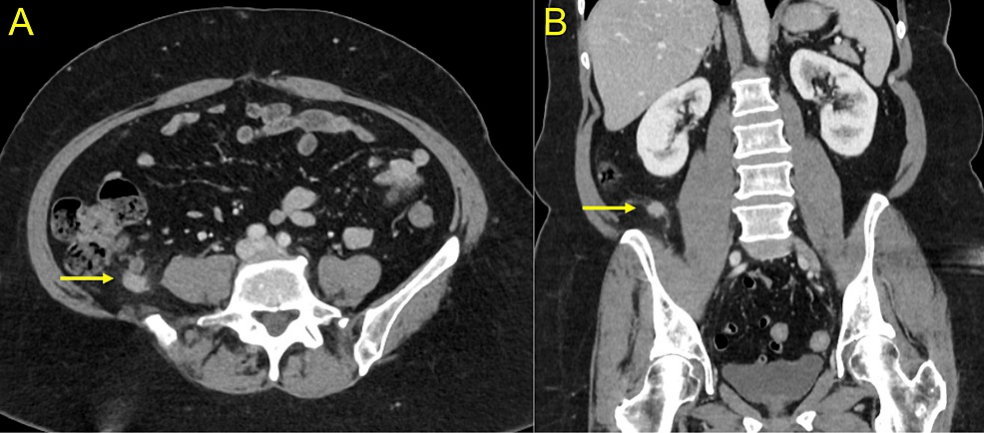
Seven-week follow-up computed tomography (CT) of the abdomen CT scan of the abdomen at the patient's seven-week follow-up visit revealed continued reduction in the size of the periappendiceal fluid collection (yellow arrows). (A) Axial view. (B) Coronal view.

All five percutaneous drains were removed within 24 days of admission, and repeat blood cultures were negative. She was transferred to our Community Living Center, an in-house short-term nursing home, for physical and occupational therapy. The surgical team continued to follow the patient. She recovered well and was discharged home after two weeks, resulting in a total hospital stay of six weeks. CT imaging at discharge revealed no fluid collections, improvement of her soft tissue myositis, and spontaneous closure of the retroperitoneal appendiceal fistula. At her three-week follow-up visit, she reported progressive functional improvement. A CT of the abdomen and pelvis revealed an asymptomatic 2 cm walled-off fluid collection abutting the tip of the appendix (Figures 4A, 4B). CT imaging of the right lower extremity revealed minimal residual edema with no recurrent abscesses. She was scheduled for a one-month follow-up visit to monitor the periappendiceal collection and right lower extremity. She remained asymptomatic, and CT imaging at that time revealed continued improvement of the right lower extremity edema and a reduction in the size of the periappendiceal fluid collection (Figures 5A, 5B).

## Discussion

Summary

This case demonstrates successful non-operative management of perforated retrocecal appendicitis with inflammation and abscesses tracking through the right thigh and into the leg. Previous case reports of perforated appendicitis with associated lower extremity soft tissue infection have described the need for urgent surgical intervention [4-12]. In our case, surgical management of the diffuse inflammatory process would have likely involved an exploratory laparotomy, right hemicolectomy with end ileostomy, and extensive debridement of the right lower extremity. The orthopedic team felt that complete surgical debridement of the extremity may have necessitated hip disarticulation, and this was not indicated. The highly morbid nature of these interventions was weighed against the patient’s clinical stability while also considering her medical comorbidities and COVID-19 positivity, the latter of which is a known risk factor for increased perioperative mortality in patients undergoing urgent or emergent procedures [13]. We felt that the risk of operative management far outweighed the benefits. Incidentally, the patient verbalized from the outset that she would not consent to surgical intervention but would agree to bedside procedures for superficial abscesses. For these reasons, we started with conservative management, and as her condition improved, it became apparent that non-operative care was effective, appropriate, and adequate.

Relevant literature

The conventional standard of care for perforated appendicitis is percutaneous drainage and interval appendectomy [14]. However, when this disease process involves retroperitoneal fistulization extending into the soft tissues of the lower extremity, prior cases described in the literature have almost exclusively required surgical intervention, unlike in our patient [4-12].

Variable outcomes have been reported after conservative therapy for complicated appendicitis and periappendiceal abscess. Simillis et al. reported that conservative treatment was associated with fewer subsequent operations and a lower incidence of bowel obstruction, secondary abscess, and wound infection [15]. In contrast, Darwazeh et al. reported that conservative management alone was associated with more complications and longer hospital stay when compared to conservative management with interval appendectomy [16]. A retrospective review by Gee et al. showed that conservative management of periappendiceal abscess was associated with a lower complication rate and shorter hospital stay compared to surgical management [17]. However, a randomized clinical trial by Mentula et al. demonstrated that non-operative care was more likely to result in abscess recurrence and to require additional intervention compared to surgical management [18].

Rationale and clinical recommendations

Our case demonstrates the efficacy of non-operative management for complicated appendicitis with extensive soft tissue involvement in a clinically stable patient. This case serves as a reminder that complicated appendicitis can present atypically with right lower extremity pain. It is important to note that if subcutaneous emphysema is identified in the setting of appendicitis, it is critical to rule out a necrotizing soft tissue infection that would necessitate urgent surgical debridement. Exam findings, hemodynamic instability, clinical judgment, and laboratory risk assessments [19] may all be useful tools in the evaluation of necrotizing soft tissue infection. In cases such as ours, patients may benefit from a multidisciplinary team, including general surgery, interventional and diagnostic radiology, infectious disease, orthopedic surgery, and vascular surgery.

## Conclusions

Thigh pain is an uncommon presenting symptom of perforated retrocecal appendicitis. The underlying mechanism seems to involve drainage from a perforated appendix into a muscle or retroperitoneal tissue, which then makes its way into the lower extremity. In our case, the process involved fistulization from the appendix into the retroperitoneum with evidence of fluid and inflammatory change extending along the femoral sheath, ultimately seeding the tissues of the right lower extremity all the way down to the foot. Multimodal imaging, including computed tomography, may be useful in elucidating the etiology of the disease process and assessing clinical progression. The decision to pursue operative management should include a multidisciplinary assessment of the patient’s hemodynamic stability, comorbidities, and response to ongoing therapy. Our patient was successfully managed with broad-spectrum antimicrobials and source control with percutaneous drainage, needle aspiration, and a single bedside procedure. With these measures, our patient improved dramatically without being subjected to the potential morbidity and mortality associated with extensive operative interventions. However, when necrotizing soft tissue infection is suspected, prompt surgical exploration is strongly recommended.

## References

[1] Campbell WB, Lee EJ, Van de Sijpe K, Gooding J, Cooper MJ (2002). A 25-year study of emergency surgical admissions. Ann R Coll Surg Engl.

[2] Guidry SP, Poole GV (1994). The anatomy of appendicitis. Am Surg.

[3] Maa J (2012). Appendicitis: the great masquerader. Arch Intern Med.

[4] Bryan J, Ashcroft J, Hudson VE, Wong KY (2021). Unusual presentation of appendicitis as soft tissue infection of the thigh. J Surg Case Rep.

[5] Funk S, Warren K, DeNapoli TS, Mitchell IC (2021). Necrotizing fasciitis of the right lower extremity from ruptured appendicitis: case report. JBJS Case Connect.

[6] Sharma SB, Gupta V, Sharma SC (2005). Acute appendicitis presenting as thigh abscess in a child: a case report. Pediatr Surg Int.

[7] van Hulsteijn LT, Mieog JS, Zwartbol MH, Merkus JW, van Nieuwkoop C (2017). Appendicitis presenting as cellulitis of the right leg. J Emerg Med.

[8] Nanavati AJ, Nagral S, Borle N (2015). Retroperitoneal perforation of the appendix presenting as a right thigh abscess. Case Rep Surg.

[9] Korsten J, Mattey WE, Bastidas J, Sicat LC (1973). Subcutaneous emphysema of the thigh secondary to ruptured diverticulum of the ascending colon. Radiology.

[10] Edwards JD, Eckhauser FE (1986). Retroperitoneal perforation of the appendix presenting as subcutaneous emphysema of the thigh. Dis Colon Rectum.

[11] Penninga L, Wettergren A (2006). Perforated appendicitis during near-term pregnancy causing necrotizing fasciitis of the lower extremity: a rare complication of a common disease. Acta Obstet Gynecol Scand.

[12] Hsieh CH, Wang YC, Yang HR, Chung PK, Jeng LB, Chen RJ (2006). Extensive retroperitoneal and right thigh abscess in a patient with ruptured retrocecal appendicitis: an extremely fulminant form of a common disease. World J Gastroenterol.

[13] Knisely A, Zhou ZN, Wu J (2021). Perioperative morbidity and mortality of patients with COVID-19 who undergo urgent and emergent surgical procedures. Ann Surg.

[14] Cheng Y, Xiong X, Lu J, Wu S, Zhou R, Cheng N (2017). Early versus delayed appendicectomy for appendiceal phlegmon or abscess. Cochrane Database Syst Rev.

[15] Simillis C, Symeonides P, Shorthouse AJ, Tekkis PP (2010). A meta-analysis comparing conservative treatment versus acute appendectomy for complicated appendicitis (abscess or phlegmon). Surgery.

[16] Darwazeh G, Cunningham SC, Kowdley GC (2016). A systematic review of perforated appendicitis and phlegmon: interval appendectomy or wait-and-see?. Am Surg.

[17] Gee D, Babineau TJ (2004). The optimal management of adult patients presenting with appendiceal abscess: "conservative" vs immediate operative management. Curr Surg.

[18] Mentula P, Sammalkorpi H, Leppäniemi A (2015). Laparoscopic surgery or conservative treatment for appendiceal abscess in adults? A randomized controlled trial. Ann Surg.

[19] Wong CH, Khin LW, Heng KS, Tan KC, Low CO (2004). The LRINEC (Laboratory Risk Indicator for Necrotizing Fasciitis) score: a tool for distinguishing necrotizing fasciitis from other soft tissue infections. Crit Care Med.

